# Daily pattern of oviposition and fecundity in *Anopheles pseudopunctipennis* Theobald, the main vector of malaria in Mexico

**DOI:** 10.1186/s12936-026-05862-8

**Published:** 2026-03-22

**Authors:** Cuauhtémoc Villarreal-Treviño, Lilia González-Cerón, Jana Celina Ríos-Delgado, Rosa Patricia Penilla-Navarro

**Affiliations:** Centro Regional de Investigación en Salud Pública, Instituto Nacional de Salud Pública, CP 30700 Cuarta Norte y 19 Calle Poniente, Tapachula, Chiapas Mexico

**Keywords:** *Anopheles pseudopunctipennis*, Pattern of oviposition, Malaria, Fecundity

## Abstract

**Background:**

*Anopheles pseudopunctipennis* is the primary malaria vector in Mexico and is widely distributed nationwide. Understanding its developmental biology is crucial for interrupting malaria transmission.

**Methods:**

This study was conducted in Tapachula, Chiapas, a city located in southeastern Mexico. Oviposition and fecundity patterns were determined in nulliparous and parous females, individually and in groups, under insectary and semi-field conditions.

**Results:**

Regardless of whether they were grouped or individual, insectary-reared or field-collected, mosquito females exhibited a primary oviposition peak between 18:00 and 20:00, followed by a gradual decline until midnight. While nulliparous females from the insectary ceased oviposition after 24:00, a small percentage of parous females continued laying eggs throughout the night, until shortly before dawn (between 24:00 and 06:00). Parous females laid more eggs than nulliparous females

**Conclusion:**

Nulliparous and parous females of *An. pseudopunctipennis* exhibited a major oviposition peak between 18:00 and 20:00, a few minutes after sunset. Oviposition activity persisted, albeit at a lower rate, until 24:00. A small subset of parous females continued ovipositing until 6:00. Parous females were observed laying more eggs than nulliparous females. Based on the high activity of *An. pseudopunctipennis*, from dusk until midnight, chemical control with pyrethroid insecticides applied by ultra-low volume (ULV) or by thermal fogging is proposed in breeding sites during this period, in areas near localities with active malaria transmission.

## Background

*Anopheles pseudopunctipennis* Theobald is one of the main malaria vectors with the widest geographical distribution in the Americas [1]. Its range extends from the southern United States (40° N) to northern Argentina (30° S) [2]. In Mexico, it is considered the primary vector of malaria, being present in most of the country, mainly above 150 m a.s.l. [3, 4]. In a study on the diversity of *Anopheles* species in Mexico, it was found to be the most abundant species (52.91%) among 13 species collected across 19 states in diverse larval habitats between 2012 and 2016 [5]. Despite its widespread distribution and importance as a vector, many aspects of *An. pseudopunctipennis****’*** biology and behavior remain unknown, due to the historical difficulty of establishing colonies at insectary [6]. Previous studies have focused on the gonotrophic cycle and survivorship [7], biting behavior and indoor resting [8], larval ecology [9], and population genetics [10, 11]. Crossbreeding experiments, cytogenetics, and DNA analysis among populations from Mexico, Peru, and Bolivia determined that there are at least two distinct species in this complex, which were provisionally named *An. pseudopunctipennis* "A" and *An. pseudopunctipennis* "B" [10, 11]. Species "A" is present in Mexico [10]. In 1998, a method to stimulate sexual behavior was developed, enabling the first successful establishment of a natural mating colony of this vector [6]. This breakthrough allows for experimental infections with *Plasmodium vivax,* revealing high susceptibility to *P. vivax* subpopulation PvSM-B, while resistant to *P. vivax* subpopulation PvSM-A in southern Mexico [12].

To further our understanding of *An. pseudopunctipennis,* a new strain was established for a year under insectary conditions to study the daily oviposition patterns of nulliparous and parous females. To assess the impact of mosquito interactions, both individual and group cage conditions were examined. Additionally, a parallel field study was conducted using wild-caught females to provide a comparative analysis.

## Methods

### Study area, mosquito collection and colony establishment

This study was conducted in Tapachula, Chiapas, a city located in southeastern México. During a week-long survey of larval ecology (from February 5 to 9, 2018)**,** 1300 *An. pseudopunctipennis* larvae of various developmental stages were collected on edge of water pools of the Coatán River in Tapachula, Chiapas, located between the parallels 14°37′ and 15°15′ north latitude; the meridians 92°09′ and 92°28′ west longitude; at an elevation of 597 m a.s.l., annual rainfall exceeds 3,000 mm, and the average annual temperature is 24.5 ± 7 ºC [13, 14]. The collection site is located at the foot of the Sierra Madre of Chiapas, characterized by rugged topography and coffee plantations. The region has a warm, humid climate with a rainy season (May–October) and a dry season (Koppen Aw). The larvae were collected and transported to the Centro Regional de Investigación en Salud Pública (CRISP) in Tapachula, Chiapas, according to standardized techniques [15]. To establish a natural mating colony, a year-long (February 2018 to January 2019) process of sexual stimulation by application of thermoperiod was employed, as previously described [6]. Insectary conditions were maintained at a 12:12 h light–dark photoperiod, 60 ± 10% relative humidity, and 25 ± 4 °C. Adult mosquitoes were identified using taxonomic keys [16].

### Daily patterns of oviposition and fecundity

After one year of establishing the new colony of *An. pseudopunctipennis*, experiments were conducted from February to June 2019 using generations F_12_-F_16_. Recently emerged adults (1–5 day-old) were placed in a 45 × 45 × 45 cm cage with males and females cohabiting for 5 days to ensure female insemination. This cage was used to stock the females for the different experiments. This procedure was carried out in this way because *An. pseudopunctipennis* females do not copulate in small cages (dimensions less than 45 × 45 × 45 cm). In all treatments, cotton pads soaked in 5% sugar water were provided on the cage.

### Female groups from the colony under insectary conditions

For each of six replicates, 100 nulliparous females were randomly taken from the stock colony and placed in a 30 × 30 × 30 cm cage. In these cages, the females were fed with sheep's blood through artificial membrane feeders. After 72 h post-feeding, the experiment began by placing an oviposition container (5 × 17 cm, height x diameter) in each cage, adding filtered water, and putting a 2.5 cm-wide strip of filter paper at the water–air interface. The experiment started at 08:00, and containers were replaced every two hours for 24 h. After 18:00, during the container change, a hand lamp was used to illuminate the cage without directly illuminating the females to avoid altering their behavior. Mosquitoes from the same cages were blood-fed again for the next gonotrophic cycle and considered parous females. The same procedure was repeated with the nulliparous females.

### Single females from the colony under insectary conditions

In the first oviposition cycle, 150 nulliparous females were obtained from the stock colony and placed in a 30 × 30 × 30 cm cage. These females were fed sheep's blood via an artificial feeder and then transferred, after 72 h post-feeding, into individual black recipient (14 × 11 cm, height x diameter) containing 250 ml of filtered water and a 2.5 cm-wide strip of filter paper. The females were monitored for 48 h and left in the same container for another day (without recording oviposition), to obtain a greater number of parous females. For the second oviposition cycle, 110 females that oviposit during their first cycle were used. For monitoring oviposition of the second gonotrophic cycle, the same procedure was used as for the first cycle.

### Field-caught female groups under semi-field conditions

Three hundred and twenty-one *An. pseudopunctipennis* were collected between 18:00 and 24:00 (in February 2019) near a horse corral, in La Concordia locality, Chiapas (15°01′05.1"N; 92°14′26.2′′ W). These recently blood-fed females were transported to the CRISP in Tapachula and held in a 45 × 45 × 45 cm mesh cage for 72 h to complete oogenesis. To study oviposition behavior under semi-field conditions, these females were divided among three cages (30 × 30 × 30 cm), with 107 mosquitoes per cage, and a cotton pad impregnated with 10% sugar was placed on top of each cage. These cages were then transferred to a quiet, shaded outdoor garden setting on the outskirts of Tapachula, surrounded by native vegetation. Oviposition containers 5 × 17 cm, height x diameter) filled with filtered distilled water and lined with filter paper were introduced into each cage. These containers were replaced every two hours for 24 h, starting at 08:00, to monitor egg-laying patterns. As these were field-caught females, their specific gonotrophic status was unknown.

### Comparative analysis of *An. pseudopunctipennis* with other *Anopheles* species

A Pubmed search was conducted to compare *An. pseudopunctipennis* oviposition pattern with that of other *Anopheles* species, using the key words: “Daily oviposition *Anopheles”* and “diel oviposition *Anopheles*”

### Statistical analysis

A Student's t-test (unpaired, with a significance level of 0.05) was used to compare mean egg production between nulliparous and parous females within groups during the 18–20 h period. The mean number of eggs in parous females was also compared between 18–20 and 20–22 h.

## Results

### Daily patterns of oviposition and fecundity

#### Female groups from the colony under insectary conditions

Nulliparous females. Six replicates of 100 nulliparous females each laid most of their eggs (92.7%) in a single, sharp peak between 18:00 and 20:00 (mean ± SD per replicate: 370.8 ± 177.2 eggs). The remaining 7.3% (174 eggs) were laid between 20:00 and 24:00. No eggs were collected after 24:00 (Table 1).

**Table 1:** Daily oviposition and egg production of nulliparous and parous grouped *Anopheles pseudopunctipennis* under insectary conditions

	1 st Cycle nulliparous^&^				2nd Cycle parous^&^				
Time (h)	No of eggs	Mean (± SD)	%		No of eggs	Mean (± SD)	%		
18–20	2225	370.8 (177.2)		92.7	6,623	1103.8 (533.1)		83.4	
20–22	151	25.2 (17.2)		6.30	901	150.2 (109.1)		11.4	
22–24	23	3.8 (4.5)		0.96	199	33.2 (26.9)		2.5	
24–02	0	0		0	24	4.0 (9.8)		0.3	
02–04	0	0		0	123	20.5 (19.4)		1.5	
04–06	0	0		0	68	11.3 (11.4)		0.8	
06–08	0	0		0	0	0		0	
08–18	0	0		0	0	0		0	
Total	2399				7938				

^&^Mean of six groups of 100 females each

Parous females. Six replicates of 100 parturient females each also showed a main peak between 18:00 and 20:00, with an egg percentage of 83.4 (mean ± SD per replicate: 1103.8 ± 533.1 eggs). Subsequently, in the following period, from 20:00 to 22:00, the number of eggs decreased significantly (mean ± SD per replicate: 150.2 ± 109.0 eggs) (*t* = 42.9356, df = 1198, *p* < 0.001), without showing an increase during the night. Unlike nulliparous females, parous females continued laying eggs from 24:00 to 6:00, accounting for an additional 2.6% of their total egg production. When comparing nulliparous females against parous, a significant increase in the number of eggs was found from 2225 to 6623 (*t* = 31.9659, df = 1198, *p* < 0.001), during a time of 18:00 to 20:00 h (Table 1).

### Single females from the colony under insectary conditions

Nulliparous females. From those laying eggs (41 of 150), 65.9% laid eggs between 18:00 and 20:00, contributing with 70% of the total egg production (2334 of 3335). During this peak, each female laid a mean ± SD of 86.4 ± 46.0 eggs. Twenty-nine percent of females continued laying eggs between 20:00 and 22:00, accounting for 24.3% of the total eggs. Only 5.1% females laid eggs between 22:00 and 24:00. No egg-laying activity was observed after 24:00 (Table 2).

**Table 2:** Daily oviposition and egg production of nulliparous and parous individual *Anopheles pseudopunctipennis* under insectary conditions

	1 st Cycle nulliparous				2nd Cycle parous			
Time (h)	*n*^&^	No of eggs	Mean (± SD)	%	*n*^&^	No of eggs	Mean (± SD)	%
18–20	27	2334	86.4 (46.0)	70.0	33	2508	76.0 (39.7)	600
20–22	12	810	67.5 (24.3)	24.3	10	653	65.3 (19.0)	15.6
22–24	2	191	63.7 (20.4)	5.7	6	416	69.3 (27.5)	9.9
24–02	0	0	0	0	3	221	73.7 (22.7)	5.3
02–04	0	0	0	0	3	246	82.0 (14.0)	5.9
04–06	0	0	0	0	2	131	65.5 (2.12)	3.1
06–08	0	0	0	0	0	0	0	0
08–18	0	0	0	0	0	0	0	0
Total	41	3335			57	4175		

^&^n = Number of oviposition occurrences

Parous females. Like nulliparous females, parous females (33 of 57) exhibited a major oviposition peak between 18:00 and 20:00, with 57.9% of females contributing 60% of total eggs (2508 of 4175). During this peak, each parous female laid an average of 76.0 ± 39.7 eggs. After the oviposition peak, between 20:00 and 22:00, 17.5% of females laid 15.6% of total eggs (653). Between 22:00 and 24:00, 14.6% of females laid 9.9% of total eggs. Between 24:00 and 6:00, eight females laid 14.3% of the total eggs (Table 2).

### Field-caught female groups under semi-field conditions

Wild females (n = 321) laid a total of 5264 eggs over 12 h (18:00 to 6:00). A distinct oviposition peak occurred between 18:00 and 20:00, shortly after dusk, with 3485 eggs laid (66.2% of the total). Following this peak, egg-laying activity continued throughout the night, accounting for the remaining 33.8% of the total egg production. No egg-laying was observed from 06:00 to 18:00 (Table 3). During the experiment, there was no mortality of females.

**Table 3:** Oviposition rate of field-caught *Anopheles pseudopunctipennis* females under semi-field conditions, in Tapachula, Chiapas

Time (h)	No. of eggs^&^	Mean (± SD)	% of oviposition
18–20	3485	1161.7 (151.3)	66.2
20–22	457	152.3 (75.5)	8.7
22–24	218	72.7 (21.4)	4.1
24–02	545	181.7 (45.1)	10.4
02–04	119	39.7 (6.0)	2.3
04–06	440	146.67 (60.0)	8.3
06–08	0	0	0
08–18	0	0	0
Total	5264		

^&^n = One hundred and seven field females were placed in 30 × 30x30 cm cages in triplicate

### Comparative analysis of the oviposition rate of *An. pseudopunctipennis* with other *Anopheles* species

According to the literature reviewed, the genus *Anopheles* exhibits exclusively nocturnal oviposition. The daily oviposition pattern varies among *Anopheles* species. *An. pseudopunctipennis* displayed a unique oviposition pattern from 18:00 to 20:00, compared to other anophelines (Table 4).

**Table 4:** Comparative Oviposition Patterns in *Anopheles* Species: including *An. pseudopunctipennis* from Chiapas, Mexico

Species	Oviposition pattern and timing (h)	Reference
*An. (Anopheles) pseudopunctipennis*	Unimodal (18 to 20)	This study
*An. (Cellia) gambiae s.s*	Unimodal (19 to 20)	[17]
*An. (Cdelia) gambiae s.l*	Bimodal (19 to 20) and (22 to 23)	[17]
*An. (Kerteszia) homunculus*	Unimodal (22 to 02)	[18]
*An. (Nyssorhynchus) oswaldoi*	Unimodal (22 to 12)	[19]
*An. (Anopheles) bellator*	Bimodal (16 to 24) and (02 to 06)	[20]
*An. (Anopheles) freeborni*	Unimodal (04 to 06)	[21]
*An. (Nyssorhynchus) albimanus*	Bimodal (18 to 20) and (04 to 06)	[22]
*An. (Nyssorhynchus) aquasalis*	Unimodal (24 to 04)	[23]

## Discussion

In the present study, we found that both single and grouped *An. pseudopunctipennis*, whether insectary-reared or field-caught, exhibited a primary oviposition peak between 18:00 and 20:00, followed by a gradual decline until midnight. Both single and grouped nulliparous females ceased egg-laying after 24:00, while a few single and grouped parous females continued laying eggs between 24:00 and 06:00.

A PubMed search revealed that *An. pseudopunctipennis* exhibits a unique daily oviposition pattern (Table 4), which is partially aligned with previous research on *An. gambiae s.s.*, showing a similar oviposition peak shortly after dusk, particularly between 19:00 and 20:00 [17]. In contrast, *An. gambiae s.l.* displayed bimodal oviposition cycles [17], unlike the pattern observed in our study.

Other anopheline species had greater differences, e.g., *An. homunculos* exhibits an oviposition peak from 22:00 to 02:00, during both the first and second gonotrophic cycles. However, during the first cycle, a subgroup of females continued laying eggs until 08:00 [18]. In *An. oswaldoi* in Trinidad, West Indies, oviposition occurred between 18:00 and 08:00, with a single oviposition peak between 22:00 and 24:00 [19]. In a study on *An. bellator* with registers every two hours, a bimodal nocturnal oviposition pattern in single females was observed. During the first gonotrophic cycle, two oviposition peaks occurred, the first between 16:00 and 24:00 and the second between 02:00 and 06:00. While in the second cycle, a similar pattern emerged, but with the second peak ending at 04:00 [20]. On the other hand, *An. freeborni* exhibits a primary oviposition peak between 04:00 and 06:00, closer to dawn. The same study indicated a primarily bimodal oviposition pattern for *An. albimanus* [21]. The geographic distribution of *An. freeborni* encompasses the western USA, where factors such as temperature, photoperiod, water availability, and blood meal availability fluctuate significantly throughout the year. These environmental variations likely influence the behavior of this species, leading to the late-night, near-dawn oviposition peak [22].

*Anopheles aquasalis*, a primary malaria vector in the Lesser Antilles, Trinidad and Tobago, eastern Venezuela and Southern Brazil, exhibits a single, late-night oviposition peak, between 24:00 and 4:00 [23]. This pattern differs from that of *An. pseudopunctipennis*, which shows a distinct peak shortly after twilight. The geographical distribution of *An. aquasalis* corresponds to tropical areas with high relative humidity. In the present study with *An. pseudopunctipennis* was carried out in southeastern Mexico, the climatic conditions correspond to a warm humid climate, with rain from May to October. The natural photoperiod in this area is around 12:12 light: dark, with twilight beginning at 18:30, and minor changes occur over the course of the year [24]. The mean temperature fluctuates around 25 ± 4 ºC [14]. Under these climatic conditions, *An. pseudopunctipennis* from Tapachula, Chiapas, has adopted egg-laying shortly after twilight, maintaining consistent timing throughout the year. Environmental factors significantly influence insect physiology, including oviposition behavior [25].

In this study, parous females laid more eggs than nulliparous females. This difference can probably be attributed to nutritional deficiencies during the larval stage and the acquisition of essential nutrients through blood feeding, which is crucial for oogenesis. Research on *An. pseudopunctipennis* has shown that 60% of nulliparous require a second meal for egg development [7, 26], a factor that was not provided in this study. The number of individual colonized females that laid eggs was low, 41 out of 100 in nulliparous females and 57 out of 100 in parous females. This may be due to the confined conditions, which could have inhibited oviposition behavior by limiting exposure to the necessary physicochemical stimuli [22].

Beyond the differences in egg output between nulliparous and parous females, these fecundity patterns have broader implications for the reproductive fitness and population dynamics of *An. pseudopunctipennis*. The higher fecundity of parous females, combined with the documented requirement of additional blood meals for oogenesis in many nulliparous females, suggests that reproductive success in this species is tightly linked to host availability. Such dependence may influence population rebound after chemical control and the persistence of transmission in endemic areas. Integrating fecundity into the interpretation of diel oviposition behavior expands the scope of this study, highlighting that interventions targeting reproductive output or gonotrophic cycles could complement adulticiding approaches and strengthen long-term vector control.

When analyzing the oviposition patterns of field females placed in semi-field conditions and with unknown parity status, a pattern similar to that observed in insectary experiments was noted: a main peak of oviposition between 18:00 and 20:00, followed by fewer eggs laid throughout the night. When comparing this experiment with those in the insectary, the results are consistent. Within this group of field females, there are nulliparous and parous females, so it can be inferred that the eggs collected between 18:00 and 24:00 were probably oviposited by both nulliparous and parous females. Between 24:00 and 06:00, eggs were mainly oviposited by parous females. In this experiment, 30x30x30 cm^3^ cages were used to ensure the collection of a greater number of eggs, since when using larger cages, gravid females are not stimulated to oviposit, due to the lack of external stimuli, mainly a combination of environmental factors such as physical, chemical (volatile chemicals), and biological factors present in the breeding sites [27].

In general terms, the daily oviposition pattern of malaria vectors can be determined by different intrinsic factors (endogenous rhythms), as well as extrinsic factors (environmental temperature, length of the photoperiod, availability of water bodies, type of vegetation, among others) [28]. When comparing the subgenus *Anopheles,* to which *An. pseudopunctipennis* belongs, with other subgenera, it was observed that there is no scheme in the oviposition pattern, indicating that the oviposition behavior is explained by ecological or intrinsic factors of the mosquito, rather than belonging to a specific subgenus [22, 28].

Most mosquitoes emerge from the pupa at dusk and remain resting in natural shelters to harden their cuticle and reach sexual maturity, a process that takes approximately 24 h. Mating also occurs during twilight hours [27]. In some species, such as *An. freeborni*, males form swarms to attract females and thus achieve copulation [29], while others, such as *An. pseudopunctipennis*, do not [6]. However, they all share a standard mating period that runs from twilight until midnight [6, 30]. In addition to these activities, female *An. pseudopunctipennis* also seek blood meals between 18:00 to midnight [31]. This study found that parous females after one blood feeding remain at rest in cages until their eggs reach full development in 3 days [7], then leave their resting places (within the cage) at twilight to oviposit mainly between 18:00 and 20:00 h. Based on the high activity of *An. pseudopunctipennis* between 18:00 and midnight, it is proposed to carry out chemical control with pyrethroid insecticides applied in ULV (ultra-low volume) or thermal fogging in breeding areas near localities with active transmission of *P. vivax*, to reduce the populations of this primary malaria vector and thus impact the number of malaria cases to reduce and ultimately eliminate malaria in endemic areas in México [32].

## Conclusions

Nulliparous and parous females of *An. pseudopunctipennis* exhibited a major oviposition peak between 18:00 and 20:00, a few minutes after sunset. Oviposition activity persisted, albeit at a lower rate, until 24:00. A small subset of parous females continued ovipositing until 6:00. Parous females were observed laying more eggs than nulliparous females. Based on the high activity of *An. pseudopunctipennis*, from dusk until midnight, chemical control of adult mosquitoes with pyrethroid insecticides applied by ultra-low volume (ULV) or by thermal fogging is proposed during this period, in areas near localities with active malaria transmission.

## Data Availability

All data generated during this study are included in this article and are available from the corresponding author.
